# Primary lung chordoma: a case report

**DOI:** 10.1186/s13000-024-01522-0

**Published:** 2024-07-03

**Authors:** Naoko Shigeta, Tetsuya Isaka, Kyoko Ono, Mio Tanaka, Tomoyuki Yokose, Hiroyuki Adachi, Wataru Usuba, Hiroyuki Ito

**Affiliations:** 1 Department of Thoracic Surgery, Kanagawa Cancer Center, 2-3-2 Nakao, Asahi, Yokohama, Kanagawa 241-8515 Japan; 2 Department of Pathology, Kanagawa Cancer Center, 2-3-2 Nakao, Asahi, Yokohama, Kanagawa 241-8515 Japan; 3 Department of Pathology, Kanagawa Children’s Medical Center, 1-138-4 Mutsukawa, Minami, Yokohama, Kanagawa 232-0066 Japan; 4 Department of Urology, St. Marianna University Yokohama Seibu Hospital, 1197-1 Yasashicyo, Asahi, Yokohama, Kanagawa 241-0811 Japan

**Keywords:** Brachyury, Lung chordoma, Testicular mixed germ-cell tumor, Isochromosome 12p, FISH

## Abstract

**Background:**

Chordoma, a rare malignant tumor arising from notochordal tissue, usually occurs along the spinal axis. Only a few published reports of primary lung chordomas exist. Herein, we present a case of primary lung chordoma and discuss important considerations for diagnosing rare chordomas.

**Case presentation:**

We report a case of primary lung chordoma in a 39-year-old male with a history of testicular mixed germ-cell tumor of yolk sac and teratoma. Computed tomography revealed slow-growing solid lesions in the left lower lobe. We performed wedge resection for suspected germ-cell tumor lung metastasis. Histologically, large round or oval cells with eosinophilic cytoplasm were surrounded by large cells with granular, lightly eosinophilic cytoplasm. Tumor cells were physaliphorous. Immunohistochemistry was positive for brachyury, S-100 protein, epithelial membrane antigen, vimentin, and cytokeratin AE1/AE3, suggesting pulmonary chordoma. Re-examination of the testicular mixed germ-cell tumor revealed no notochordal elements. Although some areas were positive for brachyury staining, hematoxylin and eosin (HE) staining did not show morphological features typical of chordoma. Complementary fluorescence in situ hybridization (FISH) of the lung tumor confirmed the absence of isochromosome 12p and 12p amplification. Thus, a final diagnosis of primary lung chordoma was established.

**Conclusions:**

In patients with a history of testicular mixed germ cell tumors, comparison of histomorphology using HE and Brachyury staining of lung and testicular tumors, and analyzing isochromosome 12p and 12p amplification in lung tumors using FISH is pivotal for the diagnosis of rare lung chordomas.

## Background

Chordomas are rare malignant tumors that arise from the remnants of the embryonic notochord. They usually occur along the spinal axis, with most tumors arising in the sacrococcygeal region (50%), spheno-occipital region (30%), or throughout the vertebrae (20%) [1]. Extra-axial chordomas, which arise at a site distant from the notochord, are extremely rare and have been reported in the ulna, tibia, pelvis, deep soft tissue of the knee, gluteus maximus, and posterior chest wall [2]. Herein, we describe a case of primary lung chordoma and discuss important considerations for diagnosing rare chordomas.

## Case presentation

**Fig. 1:**
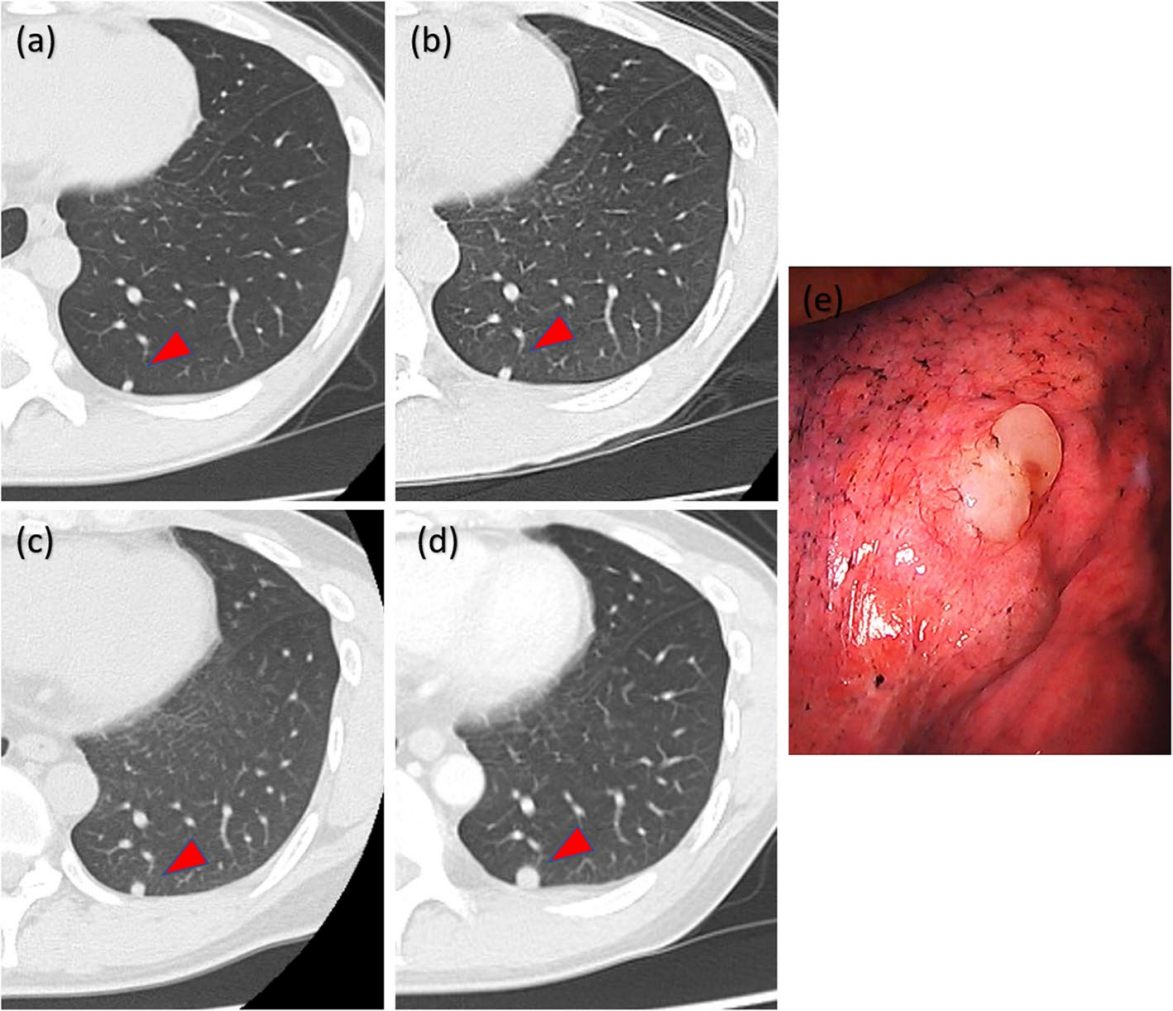
The pulmonary nodule, located in the left lower lobe on computer tomography, is a round, solid nodule with a well-defined border. **a** 13 years, **b** 8 years, **c** 3 years, and **d** 2 weeks before surgery. **e** Intraoperative findings of the pulmonary nodule

An asymptomatic 39-year-old male presented to our department for surgical resection of an 8 mm pulmonary nodule in the left lower lobe of the lung. He had a history of testicular mixed germ cell tumor comprising yolk sac and teratoma components when he was 20 years old. He also had multiple lung, liver, and bone metastases, and elevated anti-human alpha fetoprotein (AFP) (21,500 ng/mL) levels. The patient underwent inguinal orchiectomy and postoperative chemotherapy with three cycles of cisplatin, etoposide, and bleomycin (BEP). The patient achieved a complete response: all metastatic lesions disappeared on computed tomography (CT) imaging, AFP levels normalized after treatment, and no further treatment for the germ-cell tumor was required. Six years later, a 3 mm pulmonary nodule was detected in the left lower lobe (Fig. 1a). The nodule gradually increased in size, reaching 8 mm after 13 years (Fig. 1a–d). We performed a wedge resection using video-assisted thoracoscopic surgery (VATS) for suspected lung metastasis of a germ-cell tumor. Intraoperative findings revealed a white, jelly-like tumor (Fig. 1e).

**Fig. 2:**
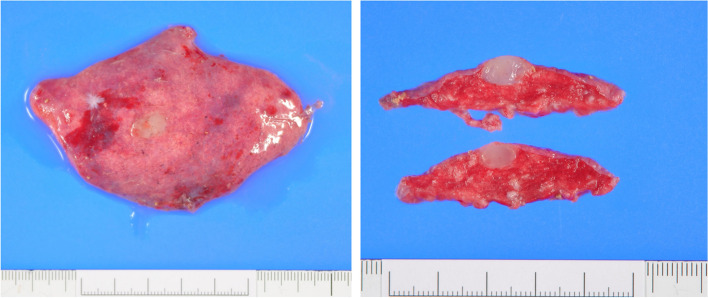
The tumor is 8 × 5 × 8 mm, well demarcated, white, and jelly-like

Macroscopically, the tumor was a well-defined milky-white, jelly-like nodule of 8 × 5 × 8 mm, located just below the pleura (Fig. 2).

**Fig. 3:**
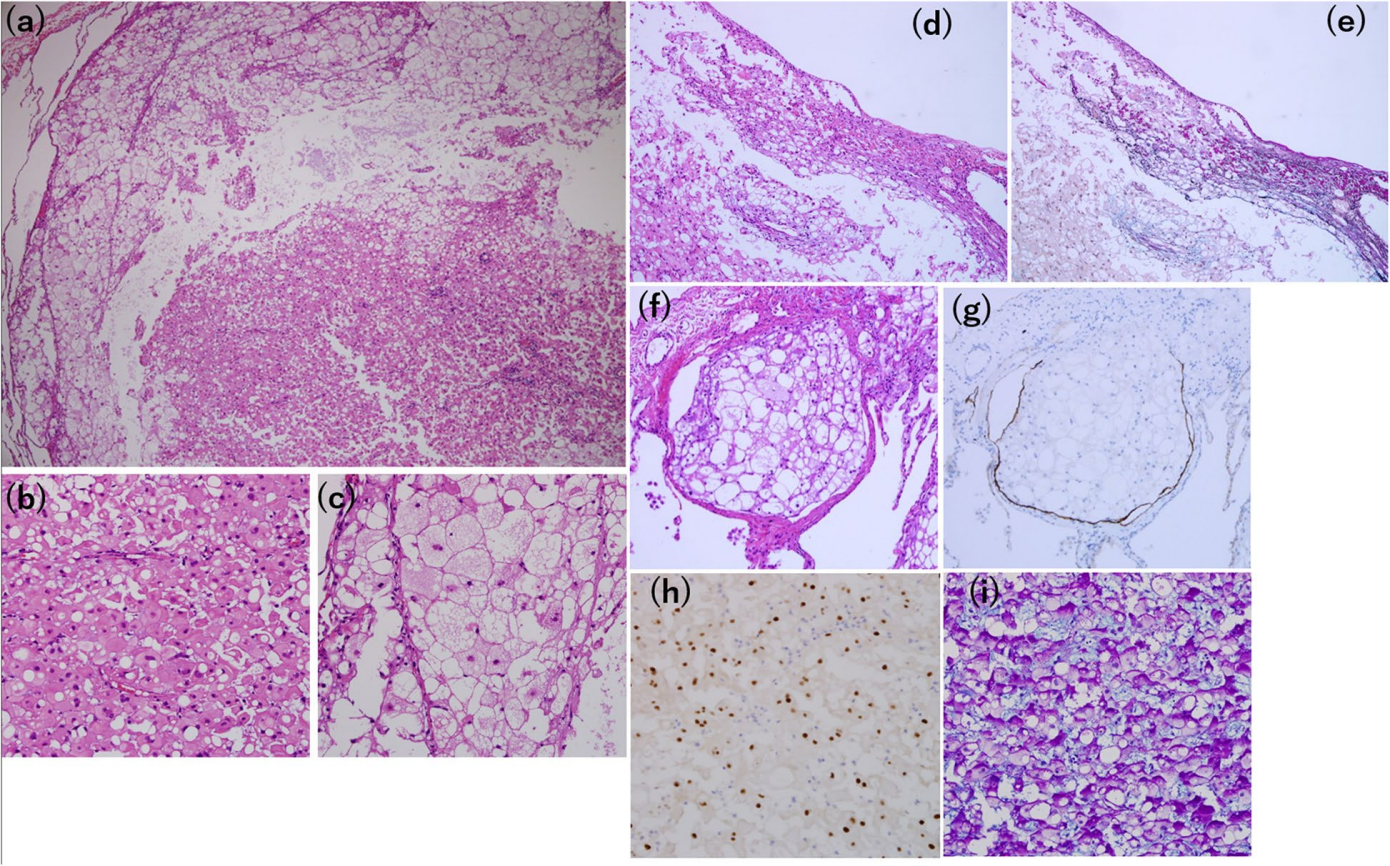
Microscopic examination revealing (**a**) solid sheets of neoplastic cells (× 40). The tumor is composed of (**b**) large round or oval cells with eosinophilic cytoplasm surrounded by (**c**) large cells with granular lightly eosinophilic cytoplasm (× 400). **d** The tumor exhibits pleural invasion and (**e**) Elastica van Gieson (EVG) staining confirms pleural disruption (× 100). **f** The tumor exhibits lymphatic invasion, and (**g**) Podoplanin (D2-40) staining confirms the presence of tumor cells in the lymphatic vessels (× 100). **h** Immunohistochemical staining shows the tumor cells, positive for brachyury (× 400). **i** Alcian blue-periodic acid-Schiff staining shows a myxoid intercellular matrix, positive for Alcian blue, surrounding the tumor cells (× 400)

Hematoxylin and eosin (HE) staining of a paraffin section revealed a tumor encapsulated with fibrous tissue that was clearly separated from the alveoli. Large round or oval cells with eosinophilic cytoplasm were surrounded by large cells with granular, lightly eosinophilic cytoplasm (Fig. 3a-c). The tumor exhibited pleural invasion (d,e) and lymphatic invasion (f,g). The tumor cells exhibited prominent cytoplasmic vacuoles resembling physaliphorous cells, and mildly hyperchromatic and pleomorphic nuclei (Fig. 3b, c). Immunohistochemistry was positive for brachyury (Fig. 3h), S-100 protein, epithelial membrane antigen, vimentin, and cytokeratin AE1/AE3. The Ki-67labeling index was 10%. These findings are consistent with chordoma. Alcian Blue-Periodic Acid Schiff (PAS) staining was used to differentiate chordoma from benign notochordal cell tumor (BNCT), and revealed an extracellular myxoid matrix not seen in BNCT (Fig. 3i).

**Fig. 4:**
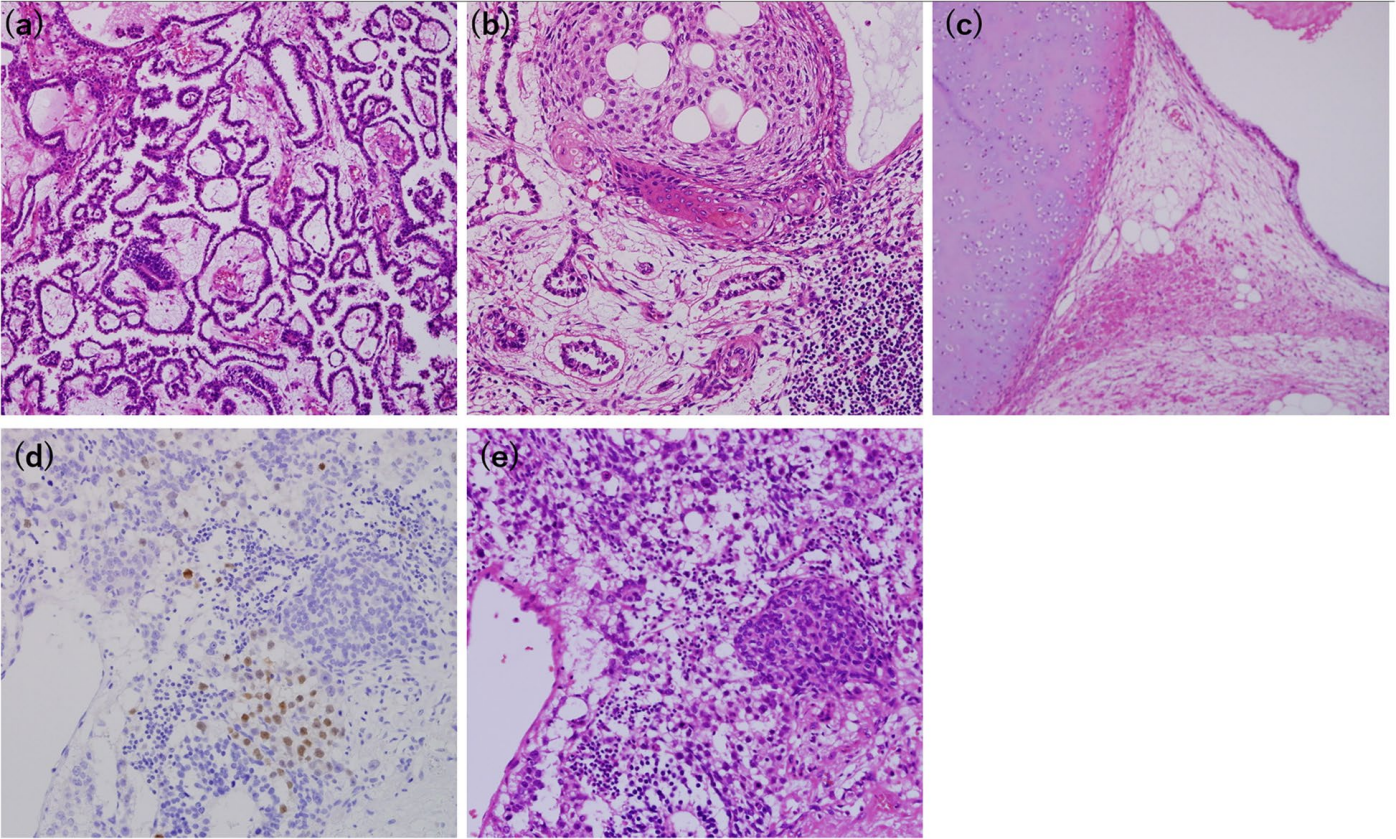
Microscopic findings of a testicular mixed germ-cell tumor of the yolk sac and teratoma (× 100). **a** In the yolk sac component of the tumor, small cells proliferate in cystic, luminal, and microcystic forms and Schiller-Duval body, which has a central vessel surrounded by tumor cells, is seen. **b** Both the yolk sac and teratoma components are seen. The squamous and glandular epithelium of the teratoma component are seen. **c** In another teratoma component, cartilage, adipocytes, and epithelium, such as gastrointestinal epithelium, are seen. **d** An area positive for brachyury. **e** HE staining of the same area as (**d**). These epithelioid cells are findings derived from teratoma, not chordoma. None of the areas positive for brachyury show notochordal cells with hematoxylin and eosin staining (× 400)

In addition, immunohistochemical analysis of AFP, Sal-like protein 4 (SALL4), PLAP, c-kit, and D2-40, performed to differentiate chordoma from lung metastases of germ-cell tumors, were all negative. Microscopic reevaluation of HE-stained paraffin sections of the testicular mixed germ-cell tumor measuring 10.5 × 7.5 × 8 cm, resected 19 years prior to the lung resection, showed no evidence of chordoma (Fig. 4a–c). Although some areas contained epithelioid cells positive for brachyury, these cells did not morphologically resemble chordoma (Fig. 4d, e).

**Fig. 5:**
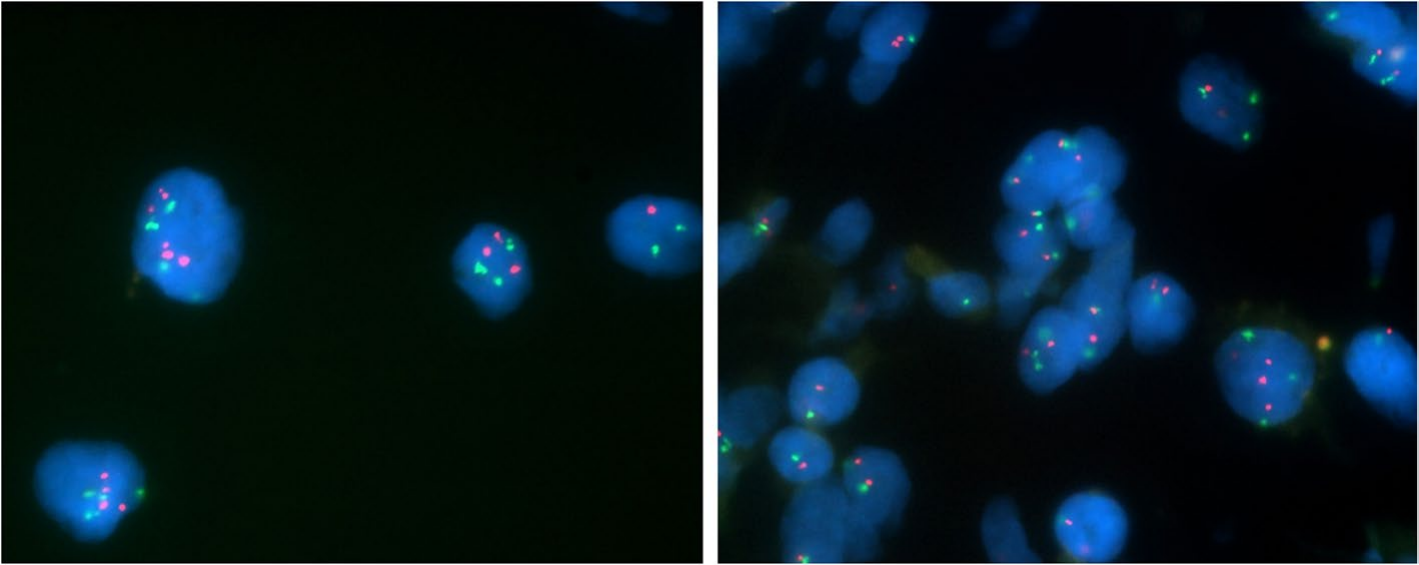
FISH analysis of the lung chordoma. Green signals represent the centromere of chromosome 12, while red signals correspond to the short arm of chromosome 12. The ratio of red to green signals was 1:1. FISH analysis showed no appearance of isochromosome 12p

Furthermore, some testicular tumor cells with a broad eosinophilic cytoplasm may have been chordoma or notochordal cells owing to their HE staining features. however, all such cells were negative for brachyury. In addition, fluorescence in situ hybridization (FISH) of the lung tumor revealed the absence of isochromosome 12p and 12p amplification, suggesting lung chordoma (Fig. 5).

The pulmonary nodule was therefore diagnosed as a primary lung chordoma. The resected specimen had negative margins and did not require additional treatment. The postoperative course was uneventful, with no recurrence at the 20-month postoperative follow-up.

## Discussion and conclusions

Chordomas are locally aggressive, slow-growing tumors that account for 1–4% of all primary malignant bone tumors and are thought to arise from notochordal cells [3, 4]. Chordomas occur mostly along the spinal axis in patients with a median age of 57 years, and are more common in males [5]. Extra-axial chordomas are rare and, to date, only a few cases of lung chordomas have been reported (Table 1). Extra-axial chordomas, including lung chordomas, are presumed to originate from a notochordal remnant with aberrant migration from the midline or from multipotent cells in the lung parenchyma [2, 6]. Kikuchi et. al. suggested that this hypothesis may also apply to the origin of extra-axial benign notochordal cell tumors (BNCTs). BNCTs could potentially serve as a precursor lesion not only for conventional axial chordomas but also for extra-axial chordoma [7].

**Table 1:** Reported cases of primary lung chordomas

Case (ref.)	Age/sex	Symptoms	Location in the lung	Number of nodules	Size	Treatment	Prognosis
1 [6]	79/F	Persistent cough and minimal right-sided chest pain	Right lower lobe	1	20 mm	Wedge resection	No recurrence for 24 months
2 [8]	79/M	Intermittent fever and dyspnea	Right lower lobe	1	73 mm	None (Diagnosed with CT-guided biopsy)	None stated
3 [9]	40/M	None	Right lower lobe, left apex and left lingular segment	8	4–23 mm	Wedge resection of 2 nodules	No recurrence and no enlargement of residual lesion for 38 months
4 [10]	61/F	None	Right apex and left lower lobe	3	Right, 18 mm; tumor size of left lobe is unknown	Segmentectomy for 3 all nodules	No recurrence for 8 months
5 [11]	59/M	Dull, exertional, episodic, substernal and right-sided chest pain	Right apex	1	36 mm	Wedge resection and proton therapy for positive margin	None stated
Our case	39/M	None	Left lower lobe	1	8 mm	Wedge resection	No recurrence for 4 months

Macroscopically, lung chordomas are usually well-demarcated, solid to cystic, with a yellow, transparent, gelatinous appearance [9]. Microscopically, HE staining of tumor sections show that intraosseous chordoma cells float in sheets, cords, or alone in an abundant myxoid stroma surrounded by a fibrous band. These cells, known as physaliphorous cells, display a vacuolated cytoplasm, and nuclei were mild-to-moderate atypia [12]. The Ki-67 labeling index in the present case (10%) is consistent with those in previous studies, ranging from 1 to 50% (median, 5%) [13]. Furthermore, chordomas are positive for brachyury, cytokeratin AE1/AE3, epithelial membrane antigen, vimentin, and S-100 protein [12, 14]. Brachyury regulates the development of notochordal cell differentiation, and positive immunohistochemistry of brachyury can differentiate chordomas from chondrosarcomas and chordoid gliomas [7]. However, benign notochordal cell tumors (BNCT) are also positive for brachyury. Several studies have attempted to distinguish chordomas from BNCT. According to these prior studies, unlike BNCT, tumor cells in chordomas have a myxoid matrix between them [7, 15]. In our case, Alcian blue-PAS staining revealed an abundant myxoid intercellular matrix. Second, according to the studies, a chordoma is encompassed by a thin fibrous membrane with a very smooth border, while a BNCT does not show any fibrous capsule formation [15]. In our case, the tumor was encapsulated by a fibrous tissue that was separated from the alveoli. Third, a previous study suggested that the nuclei of chordoma are much more atypical than those of BNCT [15]. In our case, the nuclei were mildly hyperchromatic and pleomorphic. Thus, we considered the tumor a chordoma rather than BNCT. In addition, the malignant findings of pleural and lymphatic invasion supported the diagnosis of chordoma.

Previous reports of lung chordomas have described microscopic findings similar to those of chordomas arising from notochordal tissue. The tumor cells of lung chordomas are surrounded by a fibrous capsule that separates them from the surrounding alveoli [16]. Furthermore, immunohistochemistry of lung chordomas has been reported to be positive for the same antibodies as chordomas arising from notochordal tissue [6, 8–10]. The microscopic and macroscopic findings of lung chordomas presented in this study were consistent with those previously reported. Previously published reports on lung chordomas are summarized in Table 1.

The median age was 61 (49–79) years, and the tumors ranged in size from 4 to 73 mm and were often round and solid (Table 1). Our case represents the youngest reported case, both in terms of the age at discovery and at surgery. Apart from our case study, only Ohya et al. reported long-term CT follow-up of primary lung chordoma, involving a 40-year-old patient exhibiting multiple tumors that gradually increased in size over a 14-year period [3]. In our study, the solitary tumor grew slowly and increased in diameter by 5 mm over a period of 13 years. A diagnosis of primary chordoma should therefore be considered when a solitary tumor is slow-growing and has a round, solid appearance on CT in young patients.

Careful differentiation should be made between primary lung chordomas and metastatic lung tumors of germ-cell tumors because some testicular germ-cell tumors are known to express brachyury protein [16]. Studies have reported that brachyury nuclear staining was observed in 23 out of 96 (24%) testicular germ cell tumors and it was most frequently observed in mixed tumors along with teratomas (33.3%), followed by mixed tumors (25.9%) [17]. In addition, a teratoma containing a chordoma component has been reported in a case of ovarian teratoma [18]. This case required special attention to determine whether the brachyury-positive cells found in the germ cell tumor were cells of a germ cell origin or chordoma component. This case study successfully differentiated primary lung chordoma from a metastatic lung germ-cell tumor using four methodologies. Firstly, we confirmed the absence of 12p abnormalities by FISH analysis in the resected lung chordoma. Isochromosome 12p and 12p amplification are fundamental abnormalities associated with germ cell tumors and holds significant diagnostic value in their identification [19]. Germ cell tumors can be categorized into two types: post-pubertal type with 12p abnormalities, arising from germ cell neoplasia in situ (GCNIS), and prepubertal type without 12p abnormalities, developing without GCNIS [20]. Prepubertal-type teratomas typically occur before the age of 6 years, and prepubertal-type yolk sac tumors are found in 2 to 3 cases per million children aged 0–5 years [21]. Given that the germ cell tumor occurred at 20 years of age, it was considered post-pubertal type with 12p abnormalities. In cases with 12p amplification, FISH analyses show more red signals corresponding the short arm of chromosome 12 in relation to green signals corresponding the centromere of chromosome 12. In cases with isochromosome 12p, FISH analyses show one green signal and two red signals in close proximity because the short arms of the chromosomes are close to each other. In this case of lung chordoma, the ratio of green signals to red signals were one to one in FISH analysis, and neither 12p amplification nor isochromosome 12p was not observed, making metastasis of the germ cell tumor unlikely. Second, careful reexamination of the testicular mixed germ-cell tumor revealed no chordoma component throughout the germ-cell tumor. Third, HE staining showed no morphological structures characteristic of chordoma in the areas where Brachyury staining was positive. Fourth, immunohistochemical analysis of the pulmonary nodule for germ-cell markers, including AFP, SALL4, PLAP, c-kit, and D2-40 was negative, making testicular germ-cell tumor metastasis unlikely.

The primary treatment of chordoma is surgery. No effective chemotherapy has been identified. Radiation therapy may improve local control and delay disease progression in some patients in whom complete resection is not possible [22]. McMaster et al. reported that the median survival of 361 patients with a primary chordoma was 6.29 years, with overall 5- and 10-year relative survival rates of 67.6% and 39.9%, respectively [23]. According to published reports of primary lung chordoma, four out of five cases of primary lung chordoma underwent surgery, whereas one patient refused surgery and was therefore followed up without treatment (Table 1). In our case, the tumor was completely resected using wedge resection under VATS, and there was no recurrence within 20 months postoperatively.

In conclusion, we performed complete resection of a rare primary lung chordoma that appeared as a slow-growing, solid lung tumor on CT in a patient with a history of testicular germ-cell tumor. FISH analysis of isochromosome 12p and 12p amplification of lung tumors and comparison of pathomorphology of lung and testicular tumors by HE staining and Brachyury staining are crucial in differentiating lung chordoma from metastatic lung cancer of testicular germ cell origin.

## Data Availability

Not applicable. Our manuscript does not contain any numerical data.

## Abbreviations

FISH: Fluorescence in situ hybridization
CT: Computed tomography
BNCT: Benign notochordal cell tumor
AFP: Anti-human alpha fetoprotein
SALL4: Sal-like protein 4
